# Supplementary figures and images for: Chemical composition, antioxidant and anti-inflammatory properties of Monarda didyma L. essential oil
Source: PeerJ. 2022 Nov 21;10:e14433. doi: 10.7717/peerj.14433 (PMC9686412; doi:10.7717/peerj.14433)

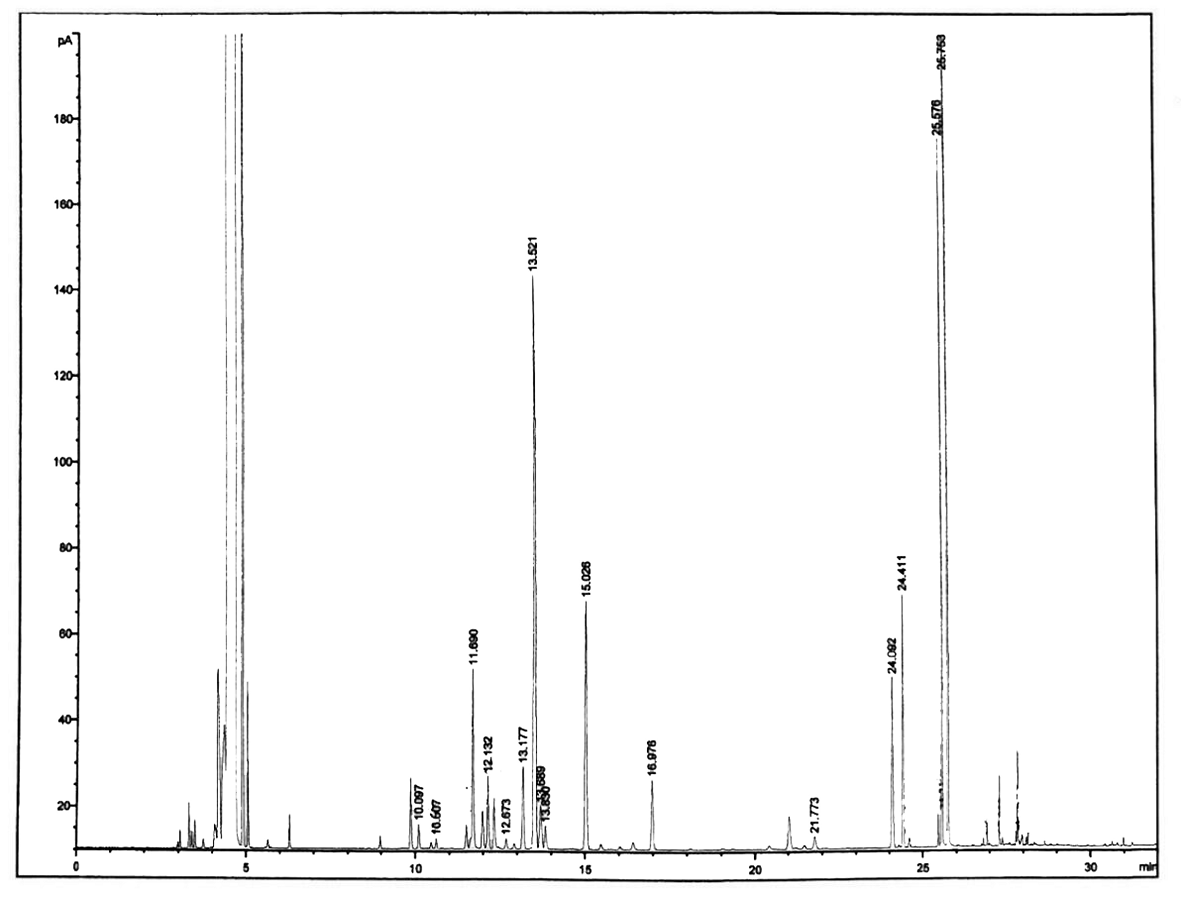

Supplement: Supplemental Information 1 — Chromatogram of Monarda didyma essential oil by GC-FID. Compounds identified by comparison of their Retention Time with that obtained with standards (Method A), are listed in Table 1. [file peerj-10-14433-s001.png]

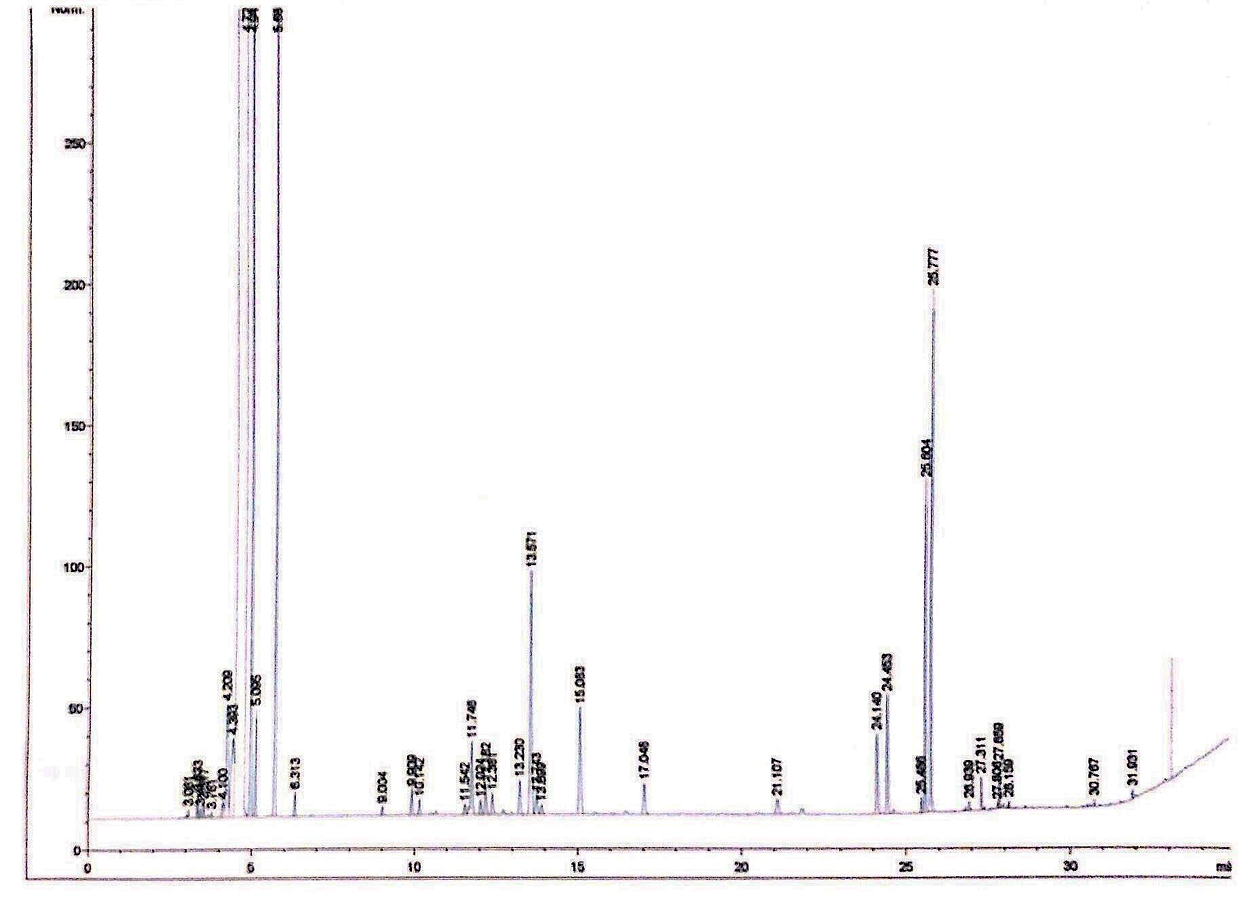

Supplement: Supplemental Information 2 — Chromatogram of Monarda didyma essential oil co-injected with the standards. Compounds identified by GC-FID in spiked essential oil (EO) with standards (Method B) and their Retention Time, are listed in Table 2. [file peerj-10-14433-s002.png]

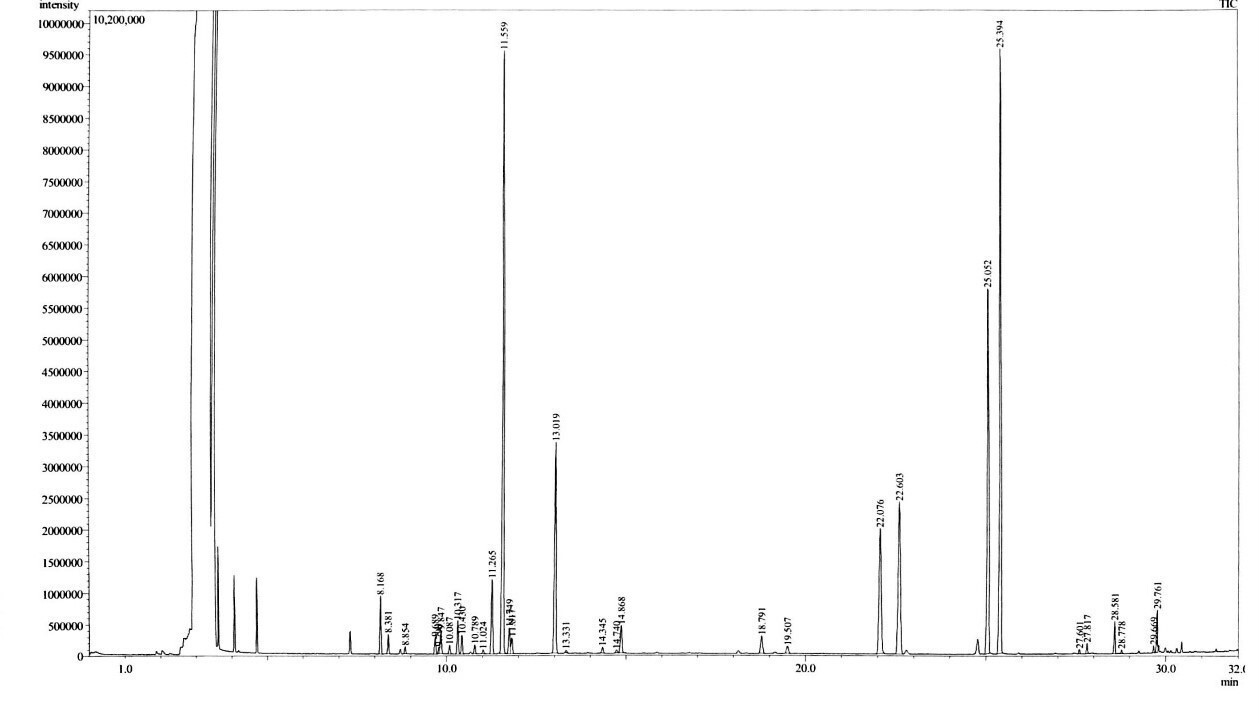

Supplement: Supplemental Information 3 — Mass chromatogram of Monarda didyma essential oil. [file peerj-10-14433-s003.jpg]
